# A comparative study between current and past Helicobacter pylori infection in terms of microalbuminuria in patients with type 2 diabetes

**DOI:** 10.1186/s12879-024-09918-5

**Published:** 2024-10-01

**Authors:** Hosam M. Ahmad, Hussein S. Al-Fishawy, Inass Shaltout, Emad A. Abd Elnaeem, Asmaa S. Mohamed, Amel E. Salem

**Affiliations:** 1 Internal medicine department, faculty of medicine, Cairo University, Cairo, Egypt; 2 Internal medicine department, Ministry of Health and population. Minia, Minia, Egypt; 3 Clinical Pathology department, Faculty of medicine, Minia University, Minia, Egypt; 4 Clinical Pharmacy and pharmacy practice department, Faculty of Pharmacy, Port said University, Port said, Egypt

**Keywords:** *H. Pylori*, Microalbuminuria, Diabetes mellitus, Infection, Renal function

## Abstract

**Background:**

The prevalence of Helicobacter pylori (*H. pylori*) infection and its potential relationship to various diseases is currently a focus of attention. The aim of this study is to investigate the association between current and past *H. pylori* infections and elevated levels of microalbuminuria in type 2 diabetic patients.

**Methods:**

Two hundred patients with type 2 diabetes mellitus were tested for the presence of *H. pylori* infection. They were divided into three groups: 52 had a current *H. pylori* infection, 38 had a past *H. pylori* infection, and 110 had no *H. pylori* infection. All study participants underwent assessments of plasma glucose levels, glycated hemoglobin (HbA1c), albuminuria levels, inflammatory markers such as erythrocyte sedimentation rate (ESR) and C-reactive protein (CRP), as well as other relevant investigations.

**Results:**

The prevalence of *H. pylori* infection (current and past) was detected in 90 out of 200 diabetic patients (45%). There was no statistically significant difference between the three groups in terms of age, diabetes duration, family history of DM, family history of hypertension, residence, or dyspeptic symptoms, indicating that current or past infection with *H. pylori* has no association with these variables. The current *H. pylori* infection group showed the highest levels of inflammatory markers, ESR and CRP, which were significantly different from those in the non-infected group (*p* = 0.013 and *p* < 0.001, respectively). The median (IQR) of albuminuria levels in the current *H. pylori* infection group, the past *H. pylori* infection group, and the non-infected group were 125 (4.8–290), 7.6 (2.4–271), and 5.1 (1.2–173), respectively. The current *H. pylori* infection group showed the highest albuminuria level, which was significantly different from that of the non-infected group (*p* = 0.001).

**Conclusion:**

There might be an association between microalbuminuria levels, general inflammatory markers (ESR and CRP), and current H. pylori infection in type 2 diabetic patients.

## Introduction

Helicobacter pylori (*H. pylori*), a spiral-shaped gram-negative bacterium, is one of the most common causes of serious chronic bacterial infections worldwide [1, 2]. *H. pylori* infection has been shown to cause chronic gastritis, peptic ulcers, gastric mucosa-associated lymphoid tissue (MALT) lymphoma, and gastric adenocarcinoma [3–6]. *H. pylori* infects nearly half of the population in the world, with strong differences between geographical areas but with consistent trends towards a decreasing incidence [7, 8]. Around 80% of individuals with *H. pylori* infection remain asymptomatic, but gastritis develops in all infected individuals, leading to unpredictable and potentially variable outcomes, as well as various levels of morbidity and mortality [9].

Helicobacter pylori infection commonly presents with a range of gastrointestinal symptoms, including dyspepsia, upper abdominal pain, bloating, nausea, and, in severe cases, peptic ulcers that can lead to bleeding [10]. *H. pylori* gastritis is a cause of dyspepsia in some patients, as evidenced by continued symptom reduction following successful *H. pylori* treatment [11].

Diagnosis of *H. pylori* infection can be achieved through non-invasive tests like the urea breath test, which detects labeled carbon dioxide in the patient’s breath after ingesting a urea solution, or the stool antigen test, which identifies *H. pylori* antigens in the stool. For more invasive approaches, endoscopy with biopsy allows direct visualization of the stomach lining, followed by histological examination or rapid urease testing [12].

Treatment typically involves a combination of antibiotics and proton pump inhibitors (PPIs) to reduce stomach acid and eradicate the bacteria. The most common regimen, triple therapy, usually includes a PPI, clarithromycin, and either amoxicillin or metronidazole. If triple therapy fails or antibiotic resistance is present, doctors may employ quadruple therapy (adding bismuth subsalicylate) or sequential therapy [13].

To confirm eradication of the bacteria after treatment, a test of cure using non-invasive techniques such as the stool antigen test or urea breath test is advised [14]. Preventive measures focus on maintaining good hygiene practices and ensuring safe food and water consumption to reduce the risk of infection [15].

**Fig. 1:**
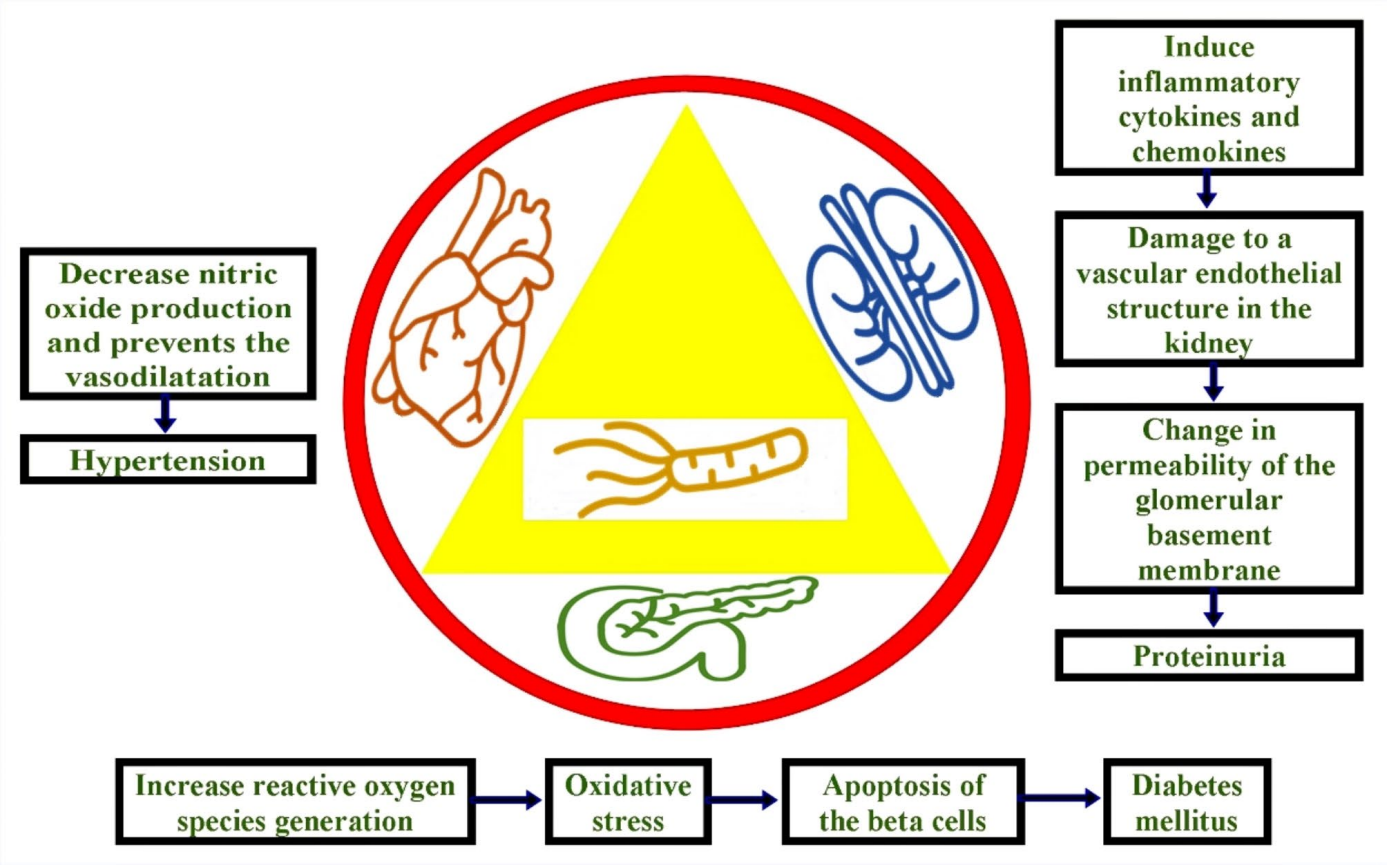
Potential effects of *H. pylori* on the heart, kidneys, and pancreas

The relationship between *H. pylori* infection and both cardiac and renal function is gaining increasing research interest. However, uncertainty remains regarding the association between *H. pylori* infection and albuminuria in type 2 diabetic patients. This lack of clarity may be due to limitations like small sample sizes, varied study methodologies, and geographical differences in *H. pylori* prevalence [16]. Figure 1 shows some of the possible ways in which *H. pylori* may affect the heart, kidneys, and pancreas [17, 18].

Microalbuminuria is defined as the presence of small amounts of albumin, a protein, in the urine. It is an early indicator of renal disease, which is a common and dangerous consequence of diabetes [19]. To treat microalbuminuria, it is important to control blood glucose levels, lower blood pressure, and follow a balanced dietary plan [20].

The study question is: In patients with type 2 diabetes (T2D), is current or past *H. pylori* infection associated with increased levels of microalbuminuria? Therefore, we aimed to investigate the association between *H. pylori* infection and microalbuminuria in these patients.

## Methods

### Study design

**Fig. 2:**
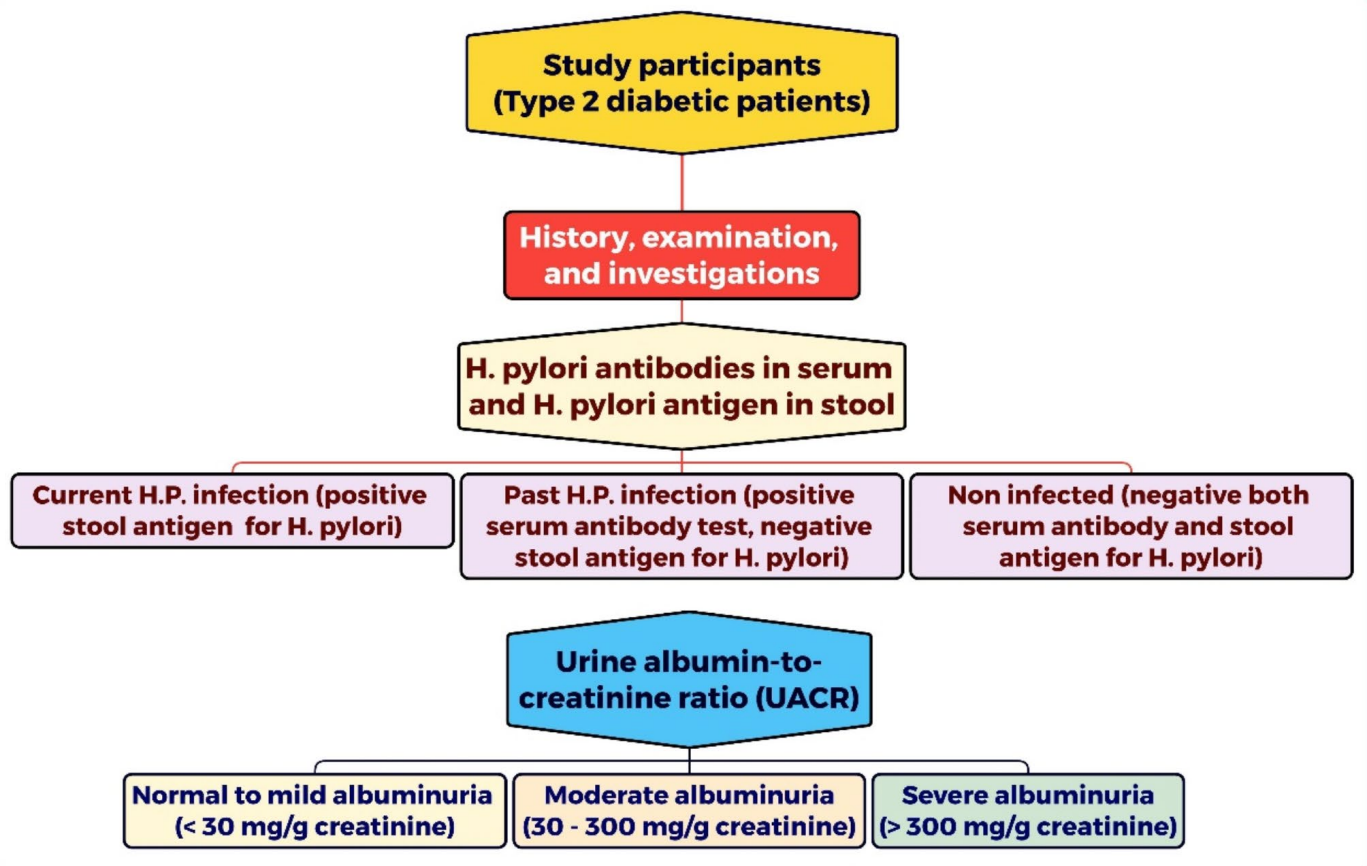
The flowchart of the study

This cross-sectional study was carried out at the internal medicine clinics of Cairo University Hospital (Kasr Al-Ainy Hospital) and Minia University Hospital, where all medical assessments were performed. The study steps are summarized in Fig. 2.

### Participants

Two hundred patients with type 2 diabetes mellitus (DM) were recruited through consecutive sampling. We divided them into three age- and gender-matched groups: (1) current *H. pylori* infection (positive stool antigen test, *n* = 52), (2) past *H. pylori* infection (positive serum antibody test, negative stool antigen test, *n* = 38), and (3) non-infected group (negative serum antibody and stool antigen tests, *n* = 110).

Inclusion criteria were adult type 2 diabetic patients aged ≥ 35 years without any severe disease.

Patients were excluded if they had a history of impaired kidney or liver function, urinary tract infections, renal stones, serum creatinine > 1.5, body mass index (BMI) ≥ 30, HbA1c ≥ 10, lupus erythematosus, rheumatoid arthritis or other systemic immunological diseases, heart failure, hypertension, any type of cancer, or had been receiving cancer therapy, antibiotics, or anti-gastric ulcer treatment in the last three months.

### Data collection and measurements

All patients underwent a medical history review and assessment of dyspepsia (discomfort or pain in the upper abdomen, often after eating or drinking), measurement of BMI, and general and systemic examinations and investigations.

### Investigations

Renal function tests (serum creatinine and urea), liver function tests (liver enzymes ALT and AST), random blood glucose level, lipid profile (total cholesterol, triglycerides, LDL, and HDL), glycated hemoglobin (HbA1c), erythrocyte sedimentation rate (ESR), C-reactive protein (CRP), and albuminuria level were assessed. *H. pylori* detection was done using stool antigen tests and anti-*H.pylori* antibodies in serum. Abdominal ultrasound was performed for all study participants.

Specific investigations included albuminuria assessment by collecting a morning urine sample to calculate the urine albumin-to-creatinine ratio (UACR). Urine albumin was measured by Micro-Albumin, a competitive ELISA test system for the quantitative measurement of human albumin in urine. The analysis was performed using ORGENTEC Diagnostika GmbH kits (Product number ORG 5MA), with a range of 1.5–400 µg/ml and functional sensitivity determined to be 0.5 µg/ml. Patients were classified according to UACR as having normal to mild albuminuria (< 30 mg/g), moderate albuminuria (30–300 mg/g), or severe albuminuria (> 300 mg/g).

The detection of *H. pylori* antigen in stool was done using the Atlas Medical Helicobacter pylori antigen test, a rapid one-step test for the detection of *H. pylori* antigen in stool samples, with a sensitivity of 95%. The detection of *H. pylori* antibodies in serum was done using Chemux Bioscience Inc. kits, an enzyme immunoassay for the quantitative determination of IgG antibodies to *H. pylori* in human serum, used for evaluating the serologic status of *H. pylori* infection in patients. The Bios *H. pylori* IgG quantitative enzyme-immunoassay test kit (catalog number: 10207) has a sensitivity of 98% and a specificity of 96%.

### Sample size

The sample size was calculated using a sample size calculator program (Power and Sample Size Calculation v3.1) with a standard error of 0.05, a power of 80%, and a confidence level of 95%, considering the prevalence of microalbuminuria in patients with type 2 diabetes.

### Outcomes and variables

The main outcome of this study was to investigate if there was an association between current or past *H. pylori* infection and the elevation of microalbuminuria levels in type 2 diabetic patients. The secondary outcomes were determining the prevalence of *H. pylori* infection in type 2 diabetic patients and comparing the study groups regarding different demographic, clinical, and laboratory markers.

### Statistical analysis

Statistical analyses were conducted using SPSS v26. Continuous variables were tested for normal distribution using the Kolmogorov-Smirnov test and presented as median (interquartile range) or mean (standard deviation), as appropriate. Comparisons of albuminuria levels between two groups were made using the Mann–Whitney test. Multiple groups were compared using the Kruskal-Wallis test for non-normally distributed data and one-way ANOVA for normally distributed data. Qualitative data were analyzed using the Chi-square test. A p-value < 0.05 indicated a significant difference. MedCalc v19 software was used to generate a receiver operating characteristic (ROC) curve.

## Results

The demographic and clinical characteristics of patients in this study are displayed in Table 1.

**Table 1:** Demographic and clinical data of the studied sample

Character	*N* = 200
**Age (year)**	
*Mean*	48.7
*(SD)*	(6.18)
**DM duration (year)**	
*Mean*	9.2
*(SD)*	(4.9)
**Gender Male/Female**	
*(n)*	57/143
*(%)*	28.5/71.5
**F.H. of D.M. (-)/(+)**	
*(n)*	145/55
*(%)*	72.5/27.5
**F.H. of HTN. (-)/(+)**	
*(n)*	164/36
*(%)*	82/18
**Residence Rural**/**Urban**	
*(n)*	154/46
*(%)*	77/23
**Dyspeptic symptoms (-)/(+)**	
*(n)*	107/93
*(%)*	53.5/46.5

SD = standard deviation; D.M. = diabetes mellitus; (-)/(+) = negative/positive; F.H. = family history; HTN. = hypertension

Table 1 shows the demographic data and clinical symptoms of the studied sample. The mean (SD) age was 48.7 (6.18) years, and the mean (SD) duration of DM was 9.2 (4.9) years. Females represented 71.5% of the sample. A positive family history of DM was found in 27.5% of cases, while a positive family history of hypertension was present in 18% of cases. 77% of the study sample was from rural areas. Dyspeptic symptoms were observed in 46.5% of the sample.

Table 2 shows the comparison between the three groups regarding demographic and clinical data. There is no statistically significant difference between the groups in age, diabetes duration, gender, family history of DM, family history of hypertension, residence, dyspeptic symptoms, systolic blood pressure, diastolic blood pressure, heart rate, and body mass index, indicating that current or past infection with *H. pylori* has no association with these variables.

**Table 2:** Comparison of demographic and clinical data between the three groups

	Current H.*P*. infection	Past H.*P*. infection	Non infected	*p*
**Age (year)**				
*Mean*	48.88	50.43	47.99	0.11
*(SD)*	(5.82)	(5.72)	(6.43)	
**D.M. duration (year)**				
*Mean*	10.3	9.62	8.56	0.113
*(SD)*	(4.74)	(4.31)	(5.17)	
**Gender Male/Female**				
*(n)*	13/39	11/27	33/77	0.8
*(%)*	25/75	28.9/71.1	30/70	
**F.H. of D.M. (-)/(+)**				
*(n)*	34/18	30/8	81/29	0.34
*(%)*	65.4/34.6	78.9/21.1	73.6/26.4	
**F.H. of HTN. (-)/(+)**				
*(n)*	43/9	31/7	90/20	0.98
*(%)*	82.7/17.3	81.6/18.4	81.8/18.2	
**Residence (Urban/ Rural)**				
*(n)*	12/40	8/30	26/84	0.95
*(%)*	23.1/76.9	21.1/78.9	23.6/76.4	
**Dyspeptic symptoms (-)/(+)**				
*(n)*	29/23	19/19	59/51	0.86
*(%)*	55.8/44.2	50/50	53.6/46.4	
**SBP (mmHg)**				
*Median*	109	106.5	109	0.38
*(IQR)*	(99.5–120)	(95–115)	(99–118)	
**DBP (mmHg)**				
*Median*	67	63	69	0.73
*(IQR)*	(57.5–80.5)	(59–76)	(58–76)	
**HR (bpm)**				
*Median*	94.5	100.5	97	0.24
*(IQR)*	(88.5-103.5)	(91–107)	(90–104)	
**BMI (kg/m** ^**2**^ **)**				
*Mean*	26.1	24.91	25.68	0.08
*(SD)*	(2.22)	(2.4)	(2.25)	

The p-value was considered significant if it was less than 0.05. H. pylori (H. P.) refers to Helicobacter pylori; FH = family history; SBP = systolic blood pressure; DBP = diastolic blood pressure; mmHg = millimeters of mercury; HR = heart rate; bpm = beats per minute; BMI = body mass index; kg/m² = kilograms per square meter. The study groups were compared using the Kruskal-Wallis test for non-normally distributed data, one-way ANOVA for normally distributed data, and the Chi-square test for qualitative data

Table 3 shows the comparison between the three groups regarding laboratory investigations. There were no significant differences between the groups for white blood cells (WBCs), red blood cells (RBCs), platelets, creatinine, alanine transaminase (ALT), aspartate transaminase (AST), or glycated hemoglobin (HbA1c). However, hemoglobin, urea level, ESR, and CRP levels all showed statistically significant differences between the groups.

**Table 3:** Comparison of laboratory investigations between the three groups

	Current H.*P*. infection	Past H.*P*. infection	Non-infected	*p*	p1	p2	p3
**WBCs (10** ^3^ **/µL)**							
*Median*	6.2	7.5	7.1	0.35	ns	ns	ns
*(IQR* **)**	(5.1–8.6)	(6.2–8.7)	(5.5–8.6)				
**RBCs (10** ^**6**^ **/µL)**							
*Mean*	4.9	5.11	5.18	0.16	ns	ns	ns
*(SD)*	(0.67)	(0.58)	(0.56)				
**Hemoglobin (g/dL)**							
*Median*	13.4	12.8	13.2	**0.019**	**0.016**	0.94	**0.007**
*(IQR)*	(12.6–14)	(11.6–13.4)	(12.3–14.2)				
**Platelets (10** ^**3**^ **/µL)**							
*Mean*	270.62	297.3	286.7	0.33	ns	ns	ns
*(SD)*	(83.7)	(103.8)	(81.7)				
**Urea (mg/dL)**							
*Median*	32	34.5	31	**0.038**	0.124	0.37	**0.01**
*(IQR)*	(24–37)	(29–40)	(25–35)				
**Creatinine (mg/dL)**							
*Mean*	0.88	0.92	0.94	0.15	ns	ns	ns
*(SD)*	(0.2)	(0.16)	(0.18)				
**ALT (U/L)**							
*Median*	18.5	21.5	19	0.68	ns	ns	ns
*(IQR)*	(14.5–30.5)	(16–27)	(16–32)				
**AST (U/L)**							
*Median*	18.5	17	18	0.42	ns	ns	ns
*(IQR)*	(12.5–22.5)	(13–21)	(14–24)				
**HbA1c (%)**							
*Mean*	7.09	7.3	6.9	0.12	ns	ns	ns
*(SD)*	(1.07)	(1.02)	(1)				
**ESR (mm/h)**							
*Median*	20.5	18.5	17	**0.023**	0.72	**0.013**	0.07
*(IQR)*	(13–28)	(15–25)	(11–21)				
**CRP (mg/dL)**							
*Median*	10.5	8	6.5	**0.001**	0.175	**< 0.001**	0.071
*(IQR)*	(6–14)	(4–13)	(2–12)				

The p-value was considered significant if it was less than 0.05; “ns” denotes non-significant. Multiple groups were compared using the Kruskal-Wallis test. Pairwise comparisons were as follows: p1 = comparison between the current H. pylori infection group and the past H. pylori infection group; p2 = comparison between the current H. pylori infection group and the non-infected group; p3 = comparison between the past H. pylori infection group and the non-infected group

The median (IQR) hemoglobin levels in the current *H. pylori* infection group, the past *H. pylori* infection group, and the non-infected group were 13.4 (12.6–14), 12.8 (11.6–13.4), and 13.2 (12.3–14.2), respectively. The past *H. pylori* infection group had the lowest hemoglobin level, which was significantly different from both the current *H. pylori* infection group and the non-infected group (*p* = 0.016 and *p* = 0.007, respectively). The median (IQR) urea levels in the current *H. pylori* infection group, the past *H. pylori* infection group, and the non-infected group were 32 (24–37), 34.5 (29–40), and 31 (25–35), respectively. The non-infected group had the lowest urea level, which was significantly different from the past *H. pylori* infection group (*p* = 0.01). The median (IQR) ESR levels in the current *H. pylori* infection group, the past *H. pylori* infection group, and the non-infected group were 20.5 (13–28), 18.5 (15–25), and 17 (11–21), respectively. The current *H. pylori* infection group had the highest ESR level, which was significantly different from the non-infected group (*p* = 0.013). The median (IQR) CRP levels in the current *H. pylori* infection group, the past *H. pylori* infection group, and the non-infected group were 10.5 (6–14), 8 (4–13), and 6.5 (2–12), respectively. The current *H. pylori* infection group had the highest CRP level, which was significantly different from the non-infected group (*p* < 0.001). This table shows a potential association between hemoglobin, urea, ESR, CRP levels, and *H. pylori* infection.

**Fig. 3:**
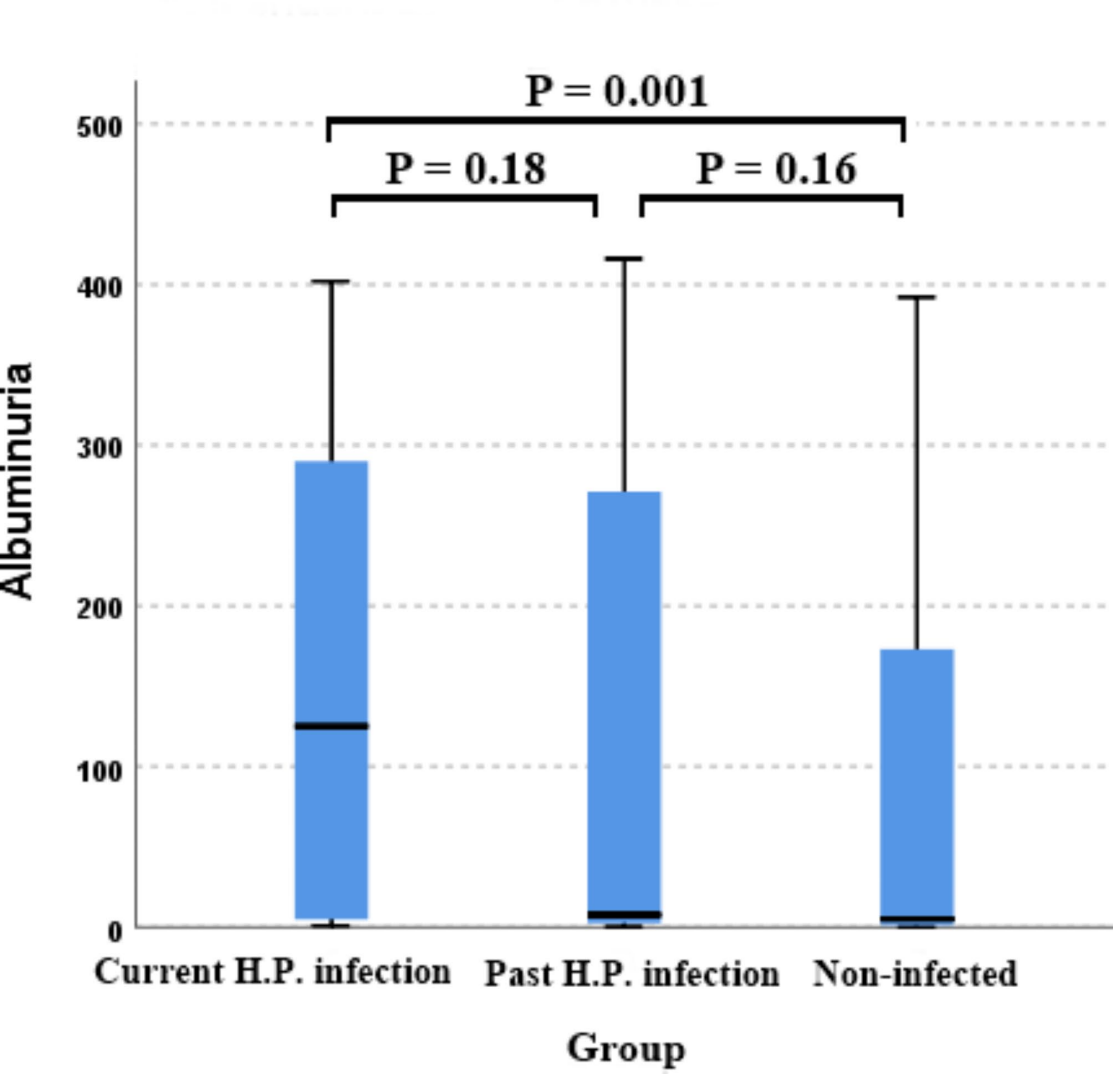
Comparison between the albuminuria levels in the three groups.The p-value was considered significant if it was less than 0.05

Figure 3 shows the comparison of albuminuria levels among the three groups. The median (IQR) albuminuria levels in the current *H. pylori* infection group, the past *H. pylori* infection group, and the non-infected group were 125 (4.8–290), 7.6 (2.4–271), and 5.1 (1.2–173), respectively. The current *H. pylori* infection group had the highest albuminuria level, which was significantly different from the non-infected group (*p* = 0.001), while there is no significant difference between the current *H. pylori* infection group and the past *H. pylori* infection group (*p* = 0.18) or between the past *H. pylori* infection group and the non-infected group (*p* = 0.16). The median level of albuminuria is highest in the current *H. pylori* infection group, followed by the past *H. pylori* infection group, and finally the non-infected group, indicating an association between current *H. pylori* infection and elevated albuminuria.

Table 4 presents the binary logistic regression analysis used to identify factors associated with the development of moderate or severe albuminuria. The analysis identifies age and positive stool antigen as significant predictors. The Beta coefficient for age is 0.342, indicating that each additional year of age increases the log-odds of developing moderate or severe albuminuria by 0.342, holding all other variables constant. With a p-value of < 0.001, this association is highly statistically significant, suggesting that older individuals are much more likely to develop moderate or severe albuminuria.

**Table 4:** Binary logistic regression analysis for predicting moderate or severe albuminuria

	Beta coefficient	S.E.	Sig.	Odds Ratio
Age	0.342	0.059	**< 0.001**	1.408
Male gender	0.339	0.482	0.482	1.403
Urban residence	0.292	0.5	0.56	1.339
Positive F.H. of D.M.	0.105	0.456	0.818	1.111
Positive F.H. of HTN.	0.232	0.538	0.667	1.261
D.M. Duration	0.076	0.047	0.108	1.079
Positive H.P. serum antibody	0.635	0.575	0.269	1.887
Positive H.P. stool antigen	1.782	0.627	**0.004**	5.941
Body mass index	0.037	0.094	0.693	1.038
Urea	0.011	0.021	0.612	1.011
Creatinine	0.201	1.151	0.861	1.223
ESR	0.038	0.031	0.222	1.039
CRP	0.004	0.04	0.929	1.004
HbA1c	0.251	0.232	0.278	1.286
Constant	-23.533-	4.208	0	0

F.H. = Family history; D.M. = Diabetes mellitus; HTN. = hypertension; H.P. = H. pylori

The Beta coefficient for a positive stool antigen test is 1.782, indicating that a positive test result significantly increases the log-odds of having moderate or severe albuminuria by 1.782, compared to those without a positive result, holding all other variables constant. The p-value of 0.004 confirms that this association is statistically significant, making a positive stool antigen a strong predictor of albuminuria. Other variables, such as gender, family history of diabetes or hypertension, diabetes duration, and various laboratory markers, were not significant predictors in this model.

**Fig. 4:**
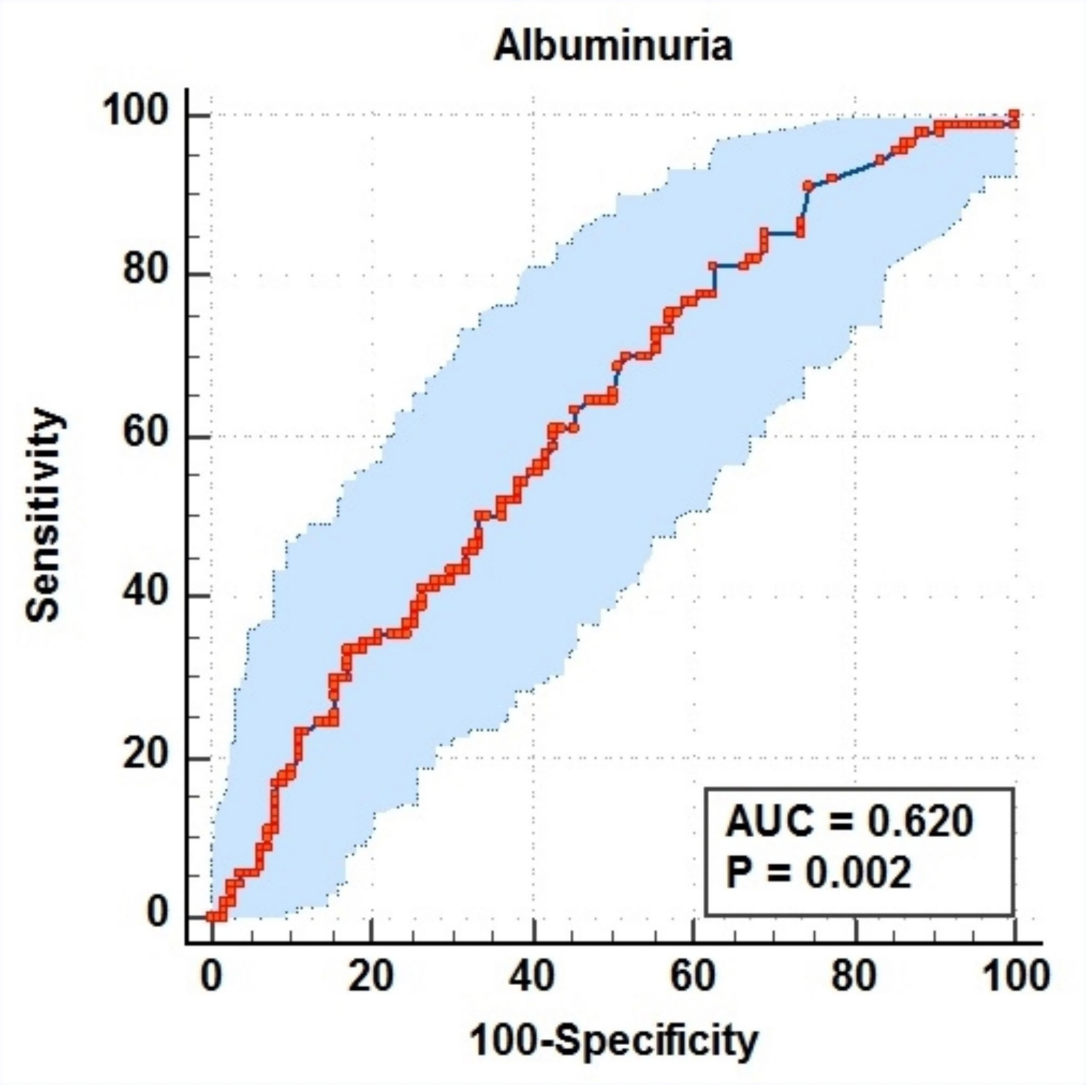
ROC curve analysis of the relationship between albuminuria levels and serum antibodies to *H. pylori*. The p-value was considered significant if it was less than 0.05

Figure 4, a receiver operating characteristic (ROC) curve, demonstrates that albuminuria levels can be used to distinguish between positive and negative serum antibodies to *H. pylori*. The ROC curve analysis indicates an area under the curve (AUC) of 0.62 (*p* = 0.002). The sensitivity, specificity, positive predictive value, and negative predictive value were 81.1%, 37.3%, 51.4%, and 70.7%, respectively. These results suggest a potential association between albuminuria levels and the presence of *H. pylori* serum antibodies.

**Fig. 5:**
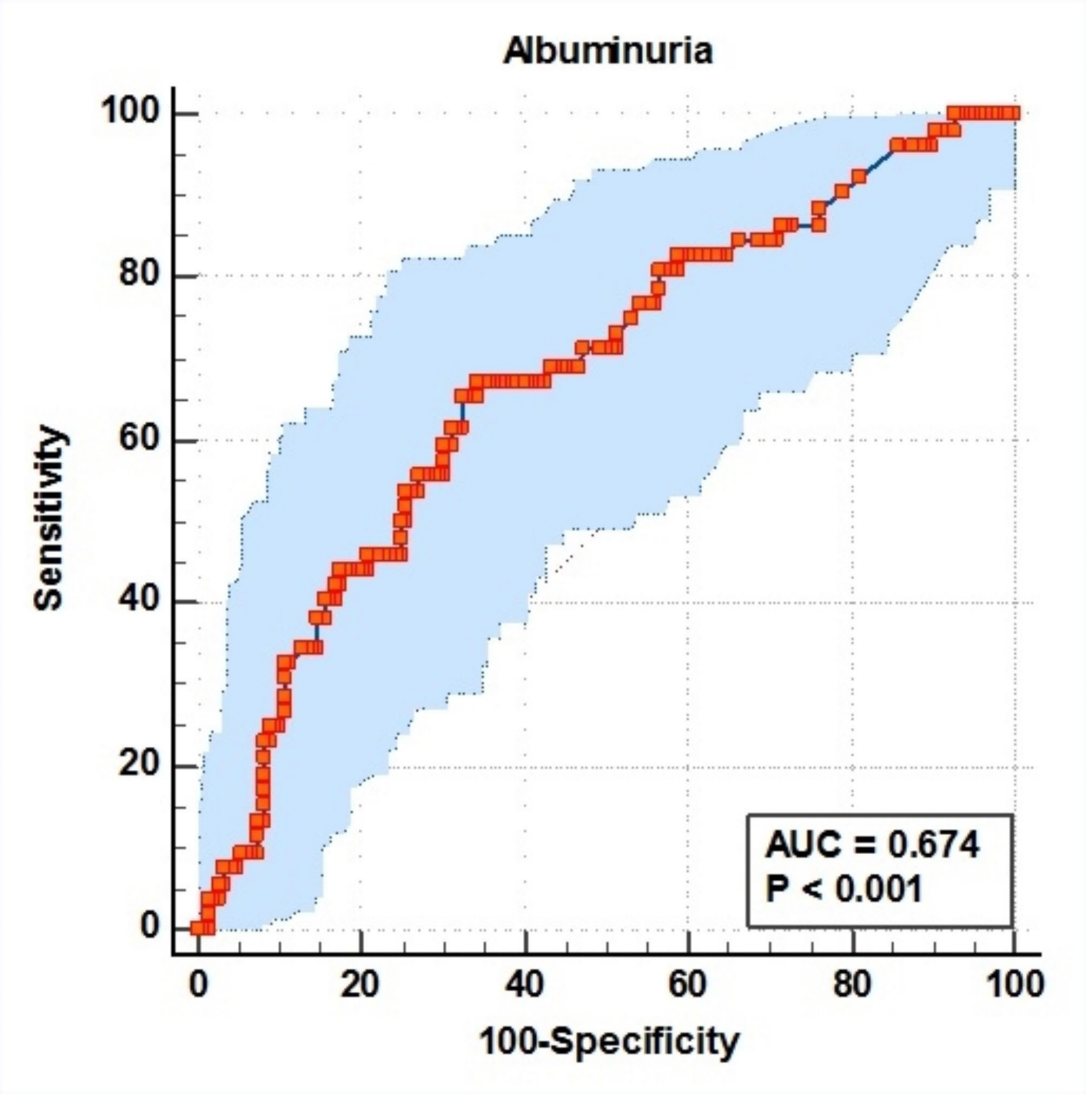
ROC curve analysis of the relationship between albuminuria levels and *H. pylori* stool antigen. The p-value was considered significant if it was less than 0.05

Figure 5 shows that albuminuria levels can be used to distinguish between positive and negative *H. pylori* stool antigens based on ROC curve analysis and its area under the curve. The sensitivity, specificity, positive predictive value, and negative predictive value were 65.38%, 67.57%, 41.5%, and 84.7%, respectively, with an area under the curve (AUC) of 0.674 (*p* < 0.001). These results suggest a potential association between albuminuria levels and the presence of *H. pylori* stool antigen.

## Discussion

The effects of *H. pylori* infection on the entire body are currently under investigation. Despite the well-established link between *H. pylori* infection and stomach pathologies, the main concern with *H. pylori* is not just its potential to cause extra-gastric disorders but also its propensity to alter disease phenotypes. According to several reports, *H. pylori*-induced chronic inflammation may be a major contributing factor to kidney diseases [21, 22]. Immunoglobulin A (IgA) nephropathy, membranous nephropathy, Henoch-Schonlein purpura nephritis, diabetic nephropathy, and other urinary disorders are strongly associated with *H. pylori*. Diseased tissues associated with these conditions have been found to contain *H. pylori* antigens [23].

The interaction between antibodies and *H. pylori* vascular wall antigens is thought to contribute to the bursting and instability of atherosclerotic plaque [24]. *H. pylori* eradication therapy is advised as a potential secondary cardiovascular preventive strategy, as *H. pylori* infection might cause endothelial dysfunction, dyslipidemia, and hyperhomocysteinemia [25]. A meta-analysis including almost 20,000 people from 26 trials revealed that *H. pylori* infection is a risk factor for myocardial infarction (MI), especially in younger patients [26]. A study of 12,836 people found that *H. pylori* infection might significantly increase the risk of carotid atherosclerosis in Chinese men under 50 years [27].

In the current study, there was a significant difference in albuminuria levels between current *H. pylori* infection, and non-infected diabetic patients. The level of albuminuria was highest in the current infection group, followed by the past infection group, and lowest in the non-infected group, suggesting a potential association between current *H. pylori* infection and increased albuminuria. Additionally, there was a significant difference in urea levels among the groups, suggesting that *H. pylori* infection may affect urea levels. However, no statistically significant difference was found among the three groups in terms of creatinine levels.

These results are consistent with another study that reported a significant association between *H. pylori* infection and the occurrence of proteinuria in type 2 diabetic patients. The study also found that proteinuria was twice as common in individuals with *H. pylori* infection compared to those without [28]. Additionally, another study found that *H. pylori*-infected individuals had significantly greater proteinuria compared to non-infected individuals, which aligns with our study’s results [29]. In line with these results, another study showed that the odds ratio of increased microalbuminuria in *H. pylori*-infected diabetic cases was 2.88 compared to non-infected cases (*p* < 0.01) [30].

In contrast to the current study, a study conducted in China, which included 22,044 adult Chinese individuals, found that the prevalence of proteinuria and the overall prevalence of chronic kidney disease (CKD) were not significantly different between the two groups studied (*H. pylori*-positive and *H. pylori*-negative subjects). After adjusting for other covariates, the odds of decreased GFR and proteinuria were not significantly different between *H. pylori*-positive and *H. pylori*-negative patients [31]. The difference in the results of this study compared to the current study may be due to the use of a single morning spot urine sample to determine proteinuria, whereas we used the urine albumin-to-creatinine ratio (UACR), which is more accurate, for the same purpose. Additionally, ethnic and national differences may result in varying outcomes due to genetic factors that influence certain diseases.

The present study revealed an association between general inflammatory markers ESR and CRP, and *H. pylori* infection. The current *H. pylori* infection group had the highest ESR level, which was significantly different from the non-infected group (*p* = 0.013). In addition, the current *H. pylori* infection group had the highest CRP level, which was significantly different from the non-infected group (*p* < 0.001).

Diabetic nephropathy is one of the most common causes of declining kidney function and the leading cause of end-stage kidney failure [32]. A new study found that patients with diabetic kidney injury had higher levels of a novel inflammatory marker, the uric acid to HDL cholesterol ratio, compared to diabetic patients without kidney injury [33]. Additionally, diabetic patients with nephropathy showed significantly increased CRP levels compared to those with type 2 diabetes mellitus who did not have nephropathy [34]. In T2D, decreased levels of the prealbumin/fibrinogen ratio may be indicative of diabetic nephropathy [35]. Additionally, the median neutrophil-to-lymphocyte ratio was found to be a predictor of good diabetes control [36].

In addition to routine tests, the hemoglobin, albumin, lymphocyte, and platelet (HALP) score, uric acid levels, C-reactive protein to serum albumin ratio (CAR), Neuregulin-4 (Nrg-4), a new adipokine released from brown adipose tissue, mean platelet volume to lymphocyte ratio, and the monocyte-to-lymphocyte ratio can serve as diagnostic predictors for diabetic nephropathy [37–44].

Elevations in inflammatory cytokines cause damage to the kidney’s vascular endothelium, allowing albumin to leak from the kidneys into the urine [45]. An inflammatory microenvironment is brought on by *H. pylori* infection, which also induces the expression of growth factors, chemokines, and other inflammatory cytokines. Moreover, *H. pylori* infection triggers the release of cytokines and vascular active substances, aggravating microvascular damage. These substances include heat shock protein (HSP), C-reactive protein (CRP), tumor necrosis factor alpha (TNF-α), and interleukins 1, 6, and 8, which elicit systemic and local immune responses [46–48]. Patients with gastric mucosal *H. pylori* infection exhibit elevated levels of CRP expression [49]. Therefore, by inducing systemic inflammation, *H. pylori* infection may cause chronic kidney injury or accelerate the loss of renal function [50].

IL-8 was one of the first cytokines found to be elevated in patients with *H. pylori* infection. It plays a role in attracting and activating inflammatory cells in the infected mucosa. IL-1α promotes angiogenesis and the proliferation of vascular endothelial cells in gastric carcinoma, particularly in children with *H. pylori*-induced inflammation. IL-1β is a key factor in triggering and amplifying inflammation, and a low IL-1β level coupled with a high TNF-α level may increase the risk of peptic ulcer disease in *H. pylori* infections. IL-10 suppresses cytotoxic inflammatory and cell-mediated immune responses, helping *H. pylori* evade the host’s immune system. Transforming Growth Factor (TGF) β acts as a continuous inflammatory mediator that aids *H. pylori* in adhering to and colonizing the host’s cells [47].

In the present cross sectional study, we described any relationship with *H. pylori* infection in type 2 diabetic patients with microalbuminuria level. The prevalence of current *H. pylori* infection was 26% and the prevalence of past *H. pylori* infection was 19%. In another study the prevalence of *H. pylori* infection has been reported to range between 30 and 80% in diabetic patients [51].

In this study, matching for gender resulted in no significant difference between current *H. pylori* infection, past *H. pylori* infection, and non-infected diabetic patients. Another study found no association between *H. pylori* infection and gender [52]. However, contrasting findings from another study reported a higher prevalence of *H. pylori* infection in males compared to females [53].

In the present study, the prevalence of *H. pylori* infection (current or past) in diabetic patients was higher in rural areas, with no significant difference regarding residence between current *H. pylori* infection, past *H. pylori* infection, and non-infected groups. This may be explained by the lower socio-economic status in rural areas compared to urban areas, limited access to healthcare, and some nutritional deficiencies. This result aligns with another study conducted in Yemen, where the infection rate was higher in rural areas (*n* = 45; 20.9%) compared to urban areas (*n* = 36; 14.7%) [54]. Several previous studies also support these findings [55]. However, a study on *H. pylori* infection and antibiotic resistance in Bhutan found that Thimphu and Punakha (urban areas) had a higher prevalence of *H. pylori* infection than rural districts [56]. Another study reported a prevalence of *H. pylori* infection at 63.67%, with no significant difference in infection rates between patients from urban and rural areas [57]. These contradictory results could be due to greater overcrowding and higher levels of environmental pollution in urban areas compared to rural areas.

In the present study, there was no significant difference regarding diabetes duration between current *H. pylori* infection, past *H. pylori* infection, and non-infected diabetic patients, indicating that *H. pylori* infection is not related to the duration of diabetes. In a systematic review and meta-analysis of the prevalence of *H. pylori* in patients with diabetes, the results showed that the prevalence of *H. pylori* had no association with the duration of diabetes (coefficient: 0.05, P: 0.935, 95% CI: -1.21, 1.32) [58]. In contrast, a case-control study about *H. pylori* infection in children with type 1 diabetes mellitus showed a correlation between the frequency of *H. pylori* and the longer duration of diabetes (*p* < 0.001) [59]. It is known that type 2 diabetes negatively affects the body’s immunity and can increase the risk of infection. However, the difference in the results of this latest study compared to ours may be due to our matching of the study participants for age, which resulted in no difference in the duration of diabetes between the study groups.

The current study showed that there was no significant difference in dyspeptic symptoms between current *H. pylori* infection, past *H. pylori* infection, and non-infected diabetic patients, suggesting that *H. pylori* infection may not be associated with dyspepsia or may be asymptomatic. This is consistent with a study that revealed around 80% of individuals with *H. pylori* infection remain asymptomatic [9, 60]. Other studies have shown that *H. pylori*-associated dyspepsia is an independent entity that resembles but is distinct from functional dyspepsia [11, 61]. The causes of dyspepsia are varied and not limited to a single factor. To confirm the role of *H. pylori* infection in the occurrence of dyspepsia, further studies are needed with adjustments for other contributing factors.

In the present study, there was no significant difference in blood pressure (systolic and diastolic) between current *H. pylori* infection, past *H. pylori* infection, and non-infected diabetic patients. Previous studies have confirmed these findings and revealed that infection with *H. pylori* is not related to any substantial changes in blood pressure [62]. To corroborate these results, other studies have shown no significant difference between two groups (infected and not infected with *H. pylori*) regarding BMI, systolic, and diastolic blood pressure [29]. This difference in results may be due to the fact that high blood pressure has multiple causes, and the duration of *H. pylori* infection was not specified in the study we conducted, which could affect the reliability of assessing its impact on blood pressure.

The current study revealed a significant difference in hemoglobin levels between the past infection group and both the current and non-infected groups. Chronic gastritis caused by *H. pylori* infection impairs iron absorption by disrupting the conversion of dietary iron from its less absorbable ferric form to the more absorbable ferrous form, resulting in anemia [63, 64]. Consistent with this finding, another study found that anemia was significantly correlated with intestinal parasite infection, living in a rural area, and *H. pylori* infection [65].

We found no significant difference in HbA1c levels between current *H. pylori* infection, past *H. pylori* infection, and non-infected diabetic patients, indicating no relationship between *H. pylori* infection and glycemic control. Supporting this, another study showed no significant difference in random blood glucose levels between *H. pylori*-infected and non-infected groups [29]. Confirming these results, other studies also concluded that *H. pylori* infection is not related to fasting glucose or glycosylated hemoglobin levels [62].

### Limitations

This study used serum antibody levels and stool antigen tests to diagnose H. pylori infection. These methods are cost-effective, easy to use, widely available, noninvasive, and have relatively high sensitivity and specificity. However, the gold standard for diagnosing H. pylori remains upper gastrointestinal endoscopy. This study did not offer longitudinal or follow-up data. Further studies with patient follow-up are recommended to confirm these results. Additionally, a larger patient sample from different geographic areas and the investigation of additional inflammatory markers would be beneficial.

## Conclusion

Current infection with Helicobacter pylori was associated with the occurrence of albuminuria in type 2 DM patients. The association between current *H. pylori* infection and general inflammatory markers (ESR and CRP) suggests a possible role of *H. pylori* infection in the inflammatory process in the human body. A past infection with *H. pylori*, however, resulted in a lower level of albuminuria than a current *H. pylori* infection. Therefore, the effect of *H. pylori* infection on albuminuria may be temporary, and treating *H. pylori* is very important for kidney health and preventing the development of diabetic nephropathy. Consequently, further studies on *H. pylori*-treated patients are recommended to confirm this.

## Data Availability

No datasets were generated or analysed during the current study.
